# Evidence of Biorealistic Synaptic Behavior in Diffusive Li-based Two-terminal Resistive Switching Devices

**DOI:** 10.1038/s41598-020-65237-0

**Published:** 2020-05-26

**Authors:** Panagiotis S. Ioannou, Evripides Kyriakides, Olivier Schneegans, John Giapintzakis

**Affiliations:** 1 Department of Mechanical and Manufacturing Engineering, University of Cyprus, 75 Kallipoleos Avenue, P.O. Box 20537, 1678 Nicosia, Cyprus; 2 Laboratoire de Génie Electrique et Electronique de Paris, CentraleSupélec, CNRS, Université Paris-Saclay, Sorbonne Université, 11 rue Joliot-Curie, 91192, Gif-sur-Yvette, France

**Keywords:** Condensed-matter physics, Electronic devices

## Abstract

Following the recent advances in artificial synaptic devices and the renewed interest regarding artificial intelligence and neuromorphic computing, a new two-terminal resistive switching device, based on mobile Li^+^ ions is hereby explored. Emulation of neural functionalities in a biorealistic manner has been recently implemented through the use of synaptic devices with diffusive dynamics. Mimicking of the spontaneous synaptic weight relaxation of neuron cells, which is regulated by the concentration kinetics of positively charged ions like Ca^2+^, is facilitated through the conductance relaxation of such diffusive devices. Adopting a battery-like architecture, using LiCoO_2_ as a resistive switching cathode layer, SiO_x_ as an electrolyte and TiO_2_ as an anode, Au/LiCoO_2_/SiO_x_/TiO_2_/p^++^-Si two-terminal devices have been fabricated. Analog conductance modulation, via voltage-driven regulation of Li^+^ ion concentration in the cathode and anode layers, along with current rectification and nanobattery effects are reported. Furthermore, evidence is provided for biorealistic synaptic behavior, manifested as paired pulse facilitation based on the summation of excitatory post-synaptic currents and spike-timing-dependent plasticity, which are governed by the Li^+^ ion concentration and its relaxation dynamics.

## Introduction

CMOS simulation of neural networks enables the implementation of artificial intelligence through machine learning^1,2^. However, it suffers from great hardware and computational cost due to the von Neumann efficiency bottleneck^3^. Novel, bioinspired computing paradigms are expected to overcome these limitations by following the blueprint of the biological brain. Contrary to von-Neumann-type systems, the astounding performance of the biological brain emanates from the highly parallel, low-energy processing and storing of information at a single node. This is achieved through the propagation of electrical and electrochemical signals between neurons, through electrical and chemical synapses, respectively, enhancing or diminishing the synaptic strength (weight) according to their preceding activity. While electrical synapses traditionally allow the bidirectional propagation of signals (action potentials), chemical synapses, which are regulated by the influx of ions such as Ca^2+^ (due to an action potential), strictly allow the unidirectional propagation of neurotransmitters from the pre-synaptic neuron to the synaptic cleft^4^. Activity-dependent plasticity of vastly interconnected neural networks enables functionalities such as long- and short-term memory, associative learning, etc., which constitute the building blocks of cognitive processes^5^.

Although still in its embryonic state, bioinspired computing appears more promising than ever before. Advancements in neural activity monitoring^6^, which will effectively lead to a higher understanding of brain functionality, along with hardware implementation and system-level simulations of neuromorphic circuits with binary and analog resistive switching devices^7–10^, constitute the foundation for the development of bioinspired computing paradigms.

While multibit information storage as synaptic weights, through the conductance modulation of such devices, enables the realization of in-memory computing and non-von Neumann architectures^11–13^, synaptic plasticity enables the direct emulation of biorealistic neural activity^14–16^. Biorealistic synaptic plasticity can be emulated by devices with transient switching behavior, such as diffusive (or second-order) memristors, which present a conductance dependence on the timing of stimulating pulses, enabling an internal timing mechanism through their inherent diffusive dynamics^17^. Mimicking the spontaneous synaptic weight relaxation of neuron cells, through the conductance relaxation mechanism of such diffusive devices, enables the implementation of short-term memory effects based on biorealistic adaptive synaptic plasticity principles.

Towards the goal of implementing such artificial synapses, resistive switching devices based on the nanoinonic diffusion of Li^+^ ions appear highly promising, both in three-terminal transistor configuration^18–22^, as well as in two-terminal vertical configuration. The latter case (top electrode/Li-based material/electrolyte/bottom electrode) can be facilitated by a combination involving Li_x_CoO_2_ as cathode material, SiO_x_ as solid electrolyte and (doped) Si as bottom electrode^23,24^. As Li^+^ ions can migrate reversibly from Li_x_CoO_2_ towards Si^25^, the Li_x_CoO_2_ conductance (which depends on the lithium content^26,27^) can be tuned between several conductance states over a range of 3–4 orders of magnitude.

However, using Si as the bottom electrode and anode, although facilitating CMOS integration, causes problems such as switching instabilities, and potentially leads to limited endurance due to a repeated high-volume expansion/contraction of Si during Li^+^ ion migration. Si anodes can exhibit up to 280% volume expansion at full lithiation^28^ which results in the degradation of the Si electrode. Optimization of the LiCoO_2_-based two-terminal architecture, through material selection and nano-engineering of the electrolyte and anode layer, has been shown to improve the switching characteristics of these devices^29^.

Taking the aforementioned limitations into consideration, a new two-terminal architecture is herein explored, based on the interjection of a thin TiO_2_ interface layer (~ 30 nm thick) between the SiO_x_ electrolyte^30^ and the Si bottom electrode. TiO_2_ has been chosen as a result of a review of recent advances in lithium-based batteries^31^, showing that Anatase and Rutile polymorphs are extremely stable under long cycling – even at high rates – with little volume expansion (~4%) upon lithium insertion/extraction^32^.

Thus, this study reports the fabrication and characterization of Li-based two-terminal resistive switching devices in the form of a Au/Li_x_CoO_2_/SiO_2_/TiO_2_/p^++^-Si stack, presenting for the first time evidence of the simultaneous manifestation of a number of memristive phenomena in the same device, like the rectification and nanobattery effects, as well as analog conductance modulation along with a range of synaptic functionalities such as spike-timing- and frequency-dependent- plasticity, which are emulated in a biorealistic manner through the device’s inherent diffusive dynamics.

## Results and Discussion

### Device fabrication and structural characterization

**Figure 1:**
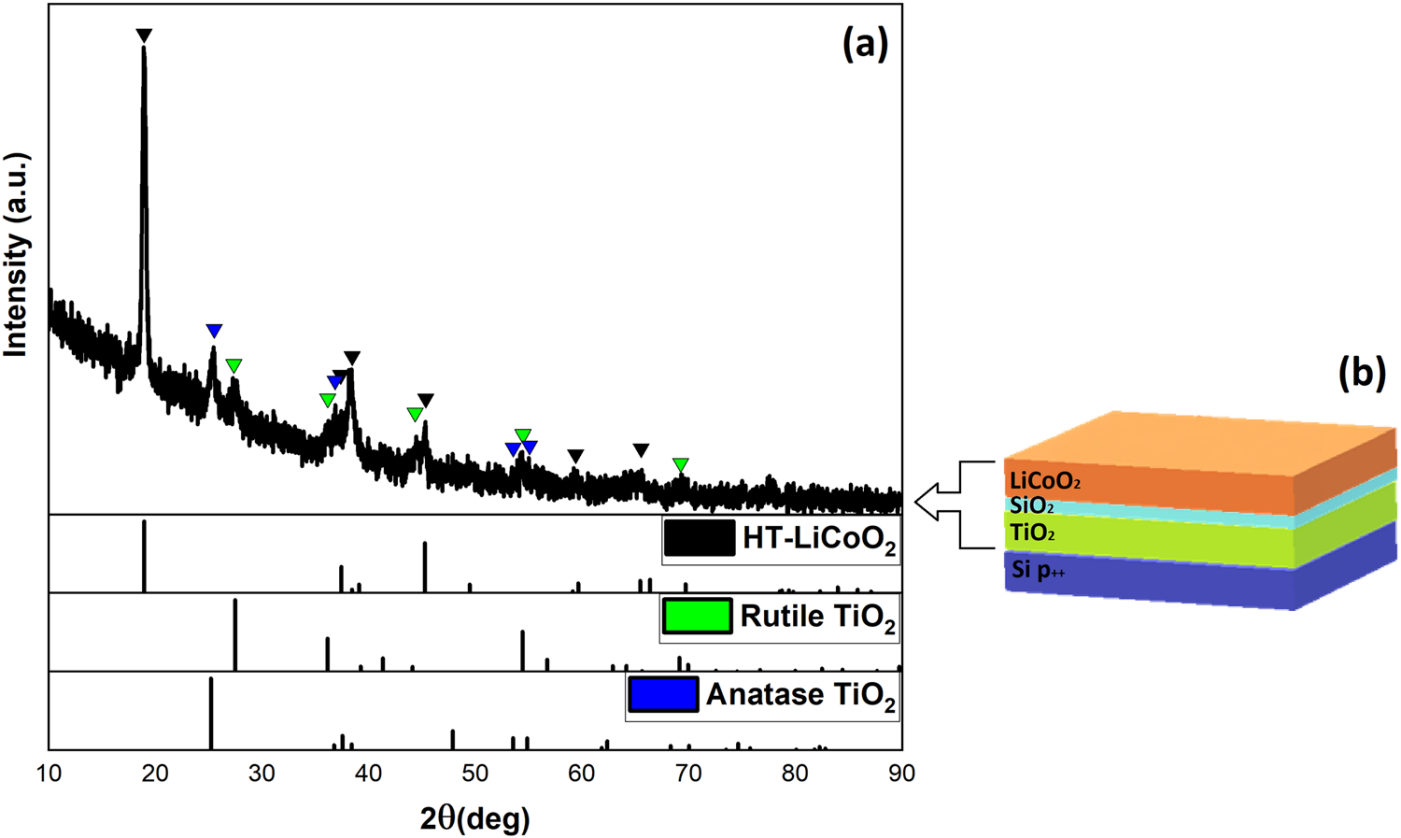
Grazing incidence X-ray diffraction pattern of the Li_x_CoO_2_/SiO_x_/TiO_2_/p^++^-Si device. The diffraction pattern indicates the presence of the mixed Anatase-Rutile TiO_2_ phases along with the nearly stoichiometric layered Li_x_CoO_2_ phase (**a**). Schematic illustration of the Li_x_CoO_2_/SiO_x_/TiO_2_/p^++^-Si multilayer stack (**b**).

Au/Li_x_CoO_2_/SiO_x_/TiO_2_/p^++^-Si devices were fabricated using a bottom-up approach, as shown in Fig. 1 (right). Pulsed laser deposition (PLD) was used for the deposition of Li_x_CoO_2_ on sputter-coated SiO_x_/Ti/p^++^-Si substrates. The deposition of nearly stoichiometric Li_x_CoO_2_ at conditions of elevated temperature (600 °C) and O_2_-rich atmosphere transforms the Ti layer to crystalline TiO_2_^33^, which effectively serves as the device anode. Figure 1 (left) shows a typical grazing incidence X-ray diffraction pattern of the Li_x_CoO_2_/SiO_x_/TiO_2_/p^++^-Si stack, indicating the presence of the mixed Anatase-Rutile TiO_2_ phases, as well as the nearly stoichiometric Li_x_CoO_2_ layer.

### DC electrical characterization

**Figure 2:**
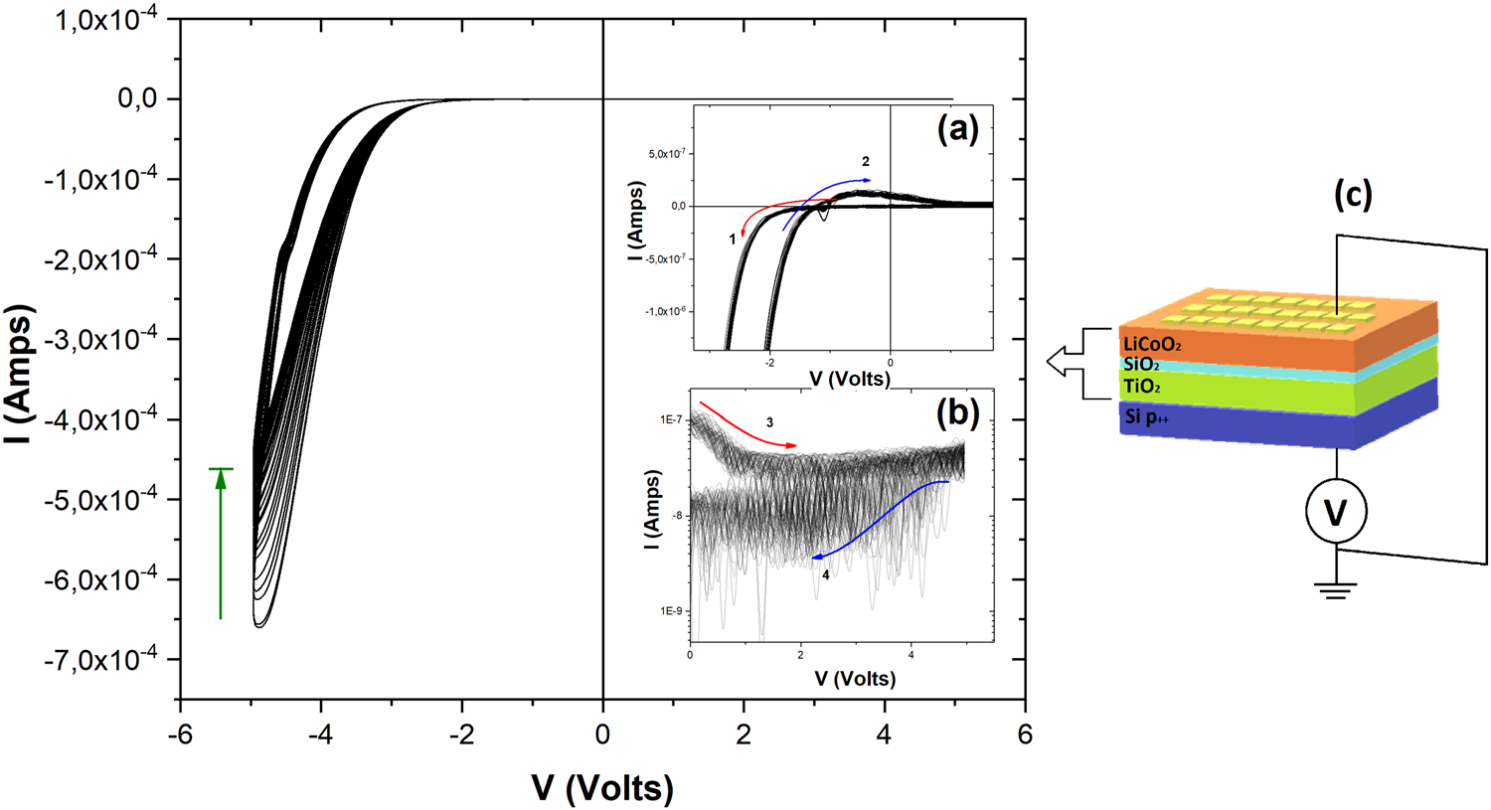
Current-Voltage (I-V) characteristic curves of the Au/Li_x_CoO_2_/SiO_x_/TiO_2_/p^++^-Si device. I-V hysteresis due to the IMT of Li_x_CoO_2_ is evident during negative biasing of the bottom Si electrode (top Au electrode: 300 × 300 μm^2^). Non-zero crossing, shown in inset (**a**), is attributed to the nanobattery effect. Current rectification during positive biasing and reset to a lower conductance can be observed in inset (**b**). Schematic of the measurement setup is also provided (**c**).

For the electrical characterization of the devices, gold top electrodes were used to cap the abovementioned multilayer stacks. In order to examine the cyclic behavior of the devices, voltage sweeps, commencing with negative bias, were applied to the devices, as shown in Fig. 2. Negative biasing, which corresponds to the application of negative voltage on the bottom Si electrode, forces Li^+^ ions to deintercalate from the Li_x_CoO_2_ cathode and migrate towards the TiO_2_ anode layer, through the SiO_x_ electrolyte. Li^+^ ion deintercalation prompts the oxidation of Co^3+^ to Co^4+^, contributing an electron to the external circuit, thus generating a hole in the Li_x_CoO_2_ valence band, which results in an insulator-metal transition (IMT)^27^. Subsequently, Li^+^ ion insertion in the TiO_2_ anode, utilizing an electron provided by the external circuit, leads to the reduction of Ti^4+^ to Ti^3+^ and the formation of lithium titanate (Li_x_TiO_2_) complexes^34^. Conversely, a subsequent positive bias will force the inserted Li^+^ ions to leave the TiO_2_ anode and re-intercalate back to the Li_1-x_CoO_2_ cathode. Thus, voltage-controlled Li^+^ ion migration leads to the reversible resistance modulation of the devices, through the IMT of the LiCoO_2_ cathode.

As can be observed in Fig. 2, initiating the DC cycling (sweep rate: 0.1 V/s) by applying a negative voltage with respect to the bottom Si electrode causes the current to increase significantly above approximately −3 V, signaling the initiation of Li^+^ ion diffusion towards the negatively charged bottom electrode. Current increase occurs smoothly and in a non-abrupt manner compared to previous approaches (e.g. Au/Li_x_CoO_2_/SiO_2_/Si stack), which presented an abrupt switch at approximately −4 V^35^. Current continues to increase as the applied voltage proceeds up to −5 V, while current hysteresis is observed upon reversal of the negative bias, towards −1.5 V. This is indicative of an enhancement of the device conductance due to the migration of Li^+^ ions from the Li_x_CoO_2_ cathode towards the TiO_2_ anode and the onset of IMT in the Li_x_CoO_2_.

Notably, with the bias still negative and progressing from −1.2 V towards 0 V, a small positive current (max: ~1.5 × 10^−7^ A) arises, resulting in a non-zero crossing in the I-V characteristic (Fig. 2, inset (a)). Non-zero crossing behavior, attributed to a nanobattery effect, emanates from the generation of an electromotive force (EMF) inside the switching device, due to charge redistribution and concentration gradients of mobile ions^36^. Positive current measured during negative bias is indicative of backpropagation of Li^+^ ions, from the TiO_2_ anode and the SiO_x_ electrolyte towards the cathode because of the aforementioned EMF^22^. The non-zero crossing I-V characteristic, because of the Li^+^ ion backpropagation (self-injection)^29^, denotes the relaxation of the conductance enhancement, highlighting the diffusive character of the devices.

Positive bias DC cycling (0 V $\leftrightarrows$ 5 V), relative to the bottom Si electrode, leads to a rectification effect resembling a diode-like behavior (Fig. 2, 1^st^ quadrant), resulting in highly asymmetric I-V characteristics. Figure 2, inset (b), indicates that the current initially decays from ~1 × 10^−7^ A (at 0 V) before reaching a value of ~4 × 10^−8^ A (above 2 V). Subsequent bias decrease from 5 V to 0 V, leads to a further decrease of the measured current (~1.5 × 10^−8^ A). These observations suggest that stimulated conductance depression, beyond the spontaneous relaxation, can be also achieved with the application of positive bias.

Consecutive DC sweeps (65 cycles), as depicted in Fig. 2, present similar characteristics. A maximum current (~−6.5 × 10^−4^ A) is measured in the first sweep (at −5 V) and subsequently fades to a constant current value (~−4.5 × 10^−4^ A) after the first 20 consecutive sweeps. The reduction in the maximum current can be attributed to partial Li^+^ ion trapping inside the SiO_x_ electrolyte during the voltage-driven Li^+^ migration^37^. After the partial Li^+^ ion loss, the device I-V characteristics gradually stabilize and continue to exhibit its distinctive traits, i.e. hysteresis, non-zero crossing nanobattery effect, rectification at positive bias and reset to a lower conductance.

It is worth noting that the devices present relatively high currents, mainly due to the large area of the top electrode (300 × 300 μm^2^). Indeed, a dependence of the maximum current on the top Au electrode area was observed (Supplementary Information, Fig. S1), which in combination with the non-abrupt switching, indicates a homogeneous rather than a filamentary switching mechanism. Such a dependence on the top electrode area can be promising for the reduction of current levels, as well as more power efficient switching with device scaling^25^.

Leakage currents during potentiation/depression and readout can result in cross-talk effects between the switching elements in a crossbar array, requiring the integration of selector diode devices between write/read lines. Inherent rectification of the investigated two-terminal device can effectively suppress leakage currents potentially rendering selector devices obsolete^38^.

Emerging evidence suggest that coexisting chemical and heterotypic electrical synapses, between premotor “command” interneurons and downstream motor neurons, interact to cooperatively modulate the synaptic strength. Furthermore, unidirectional signal transmission from heterotypic electrical synapses, achieved through current rectification, has been shown to amplify the chemical transmission (strictly unidirectional) at functionally mixed electrical-chemical synapses^39^. The inherent unidirectional signal propagation of the rectifying Au/Li_x_CoO_2_/SiO_x_/TiO_2_/p^++^-Si devices presented, in combination with the transient conductance enhancement, could potentially enable the emulation of chemical and rectifying electrical (heterotypic) synaptic behavior.

### Analog conductance modulation

**Figure 3:**
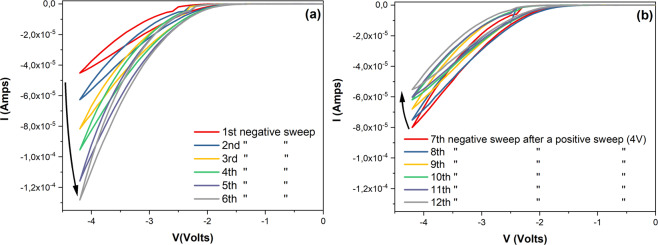
Analog conductance modulation. Consecutive sweeps with solely negative biases (**a**) results in the progressive enhancement of the Au/Li_x_CoO_2_/SiO_x_/TiO_2_/p^++^-Si conductance (top Au electrode: 300×300 μm^2^). Cumulative analog conductance enhancement was partially reversed (**b**), when a positive sweep was interjected between the subsequent six negative sweeps (sweep rate: 0.1 V/s). Positive sweeps are omitted for clarity.

Progressive enhancement and depression of the device conductance was observed, as shown in Fig. 3(a,b). Sequential negative cycling, progressively potentiated the devices to continuously higher conductance states.

This was observed when the Au/Li_x_CoO_2_/SiO_x_/TiO_2_/p^++^-Si devices were cycled with solely negative biases between 0 V and −4.2 V. When six successive negative sweeps [(0 V $\leftrightarrows$ −4.2 V) x6] were applied on the device, I-V curves with increasing hysteresis and maximum currents were induced, as shown in Fig. 3(a). This behavior, indicative of a cumulative analog conductance enhancement, was reversed when a positive sweep was interjected between each of the subsequent six negative sweeps [(4 V $\leftrightarrows$ −4.2 V) x6], as shown in Fig. 3(b).

Although consecutive negative sweeps clearly result in a progressively enhanced conductance, partial overlapping of I-V cycles indicates that the device has a diffusive, i.e. conductance relaxation, attribute (Fig. 3(a)). This is also suggested by the non-zero crossing I-V characteristics discussed previously. Additionally, the depression sweeps are not directly complementary to the corresponding potentiation sweeps (Fig. 3(b)), mainly due to the characteristic asymmetry and the diffusive attribute of the device. This behavior, of continuous potentiating or depressing during consecutive voltage stimulation, at negative or positive bias, respectively, is attributed to the motion of Li^+^ ions in the presence of an electric field. At negative bias greater than ~−2 V, Li^+^ ions migrate towards the bottom Si electrode and are inserted in the TiO_2_ anode, resulting in a mixed ionic-electronic current, while the observed hysteresis in the I-V curves is related to the conductance increase because of the induced IMT in the (now Li-deficient) Li_x_CoO_2_ layer. Measured current at negative bias has an ionic contribution coming from the moving Li^+^ ions in the device and an electronic contribution coming from the concurrent redox reactions (in the anode and cathode, respectively) and the electron transport through the device due to the induced IMT. Consecutive negative cycling (Fig. 3(a)) leads to an increasingly higher degree of Li^+^ depletion, hence the progressive enhancement of the device conductance with recurrent negative cycles. On the other hand, the interjection of positive cycles (Fig. 3(b)) leads to the stimulated extraction of Li^+^ ions from the TiO_2_ anode and their re-intercalation in the previously depleted Li_x_CoO_2_ cathode, reversing the analog conductance enhancement. This reversal is attributed to the stimulated and progressive inversion of the IMT in the Li_x_CoO_2_, which increases the cathode resistance again.

### Pulsed characterization

**Figure 4:**
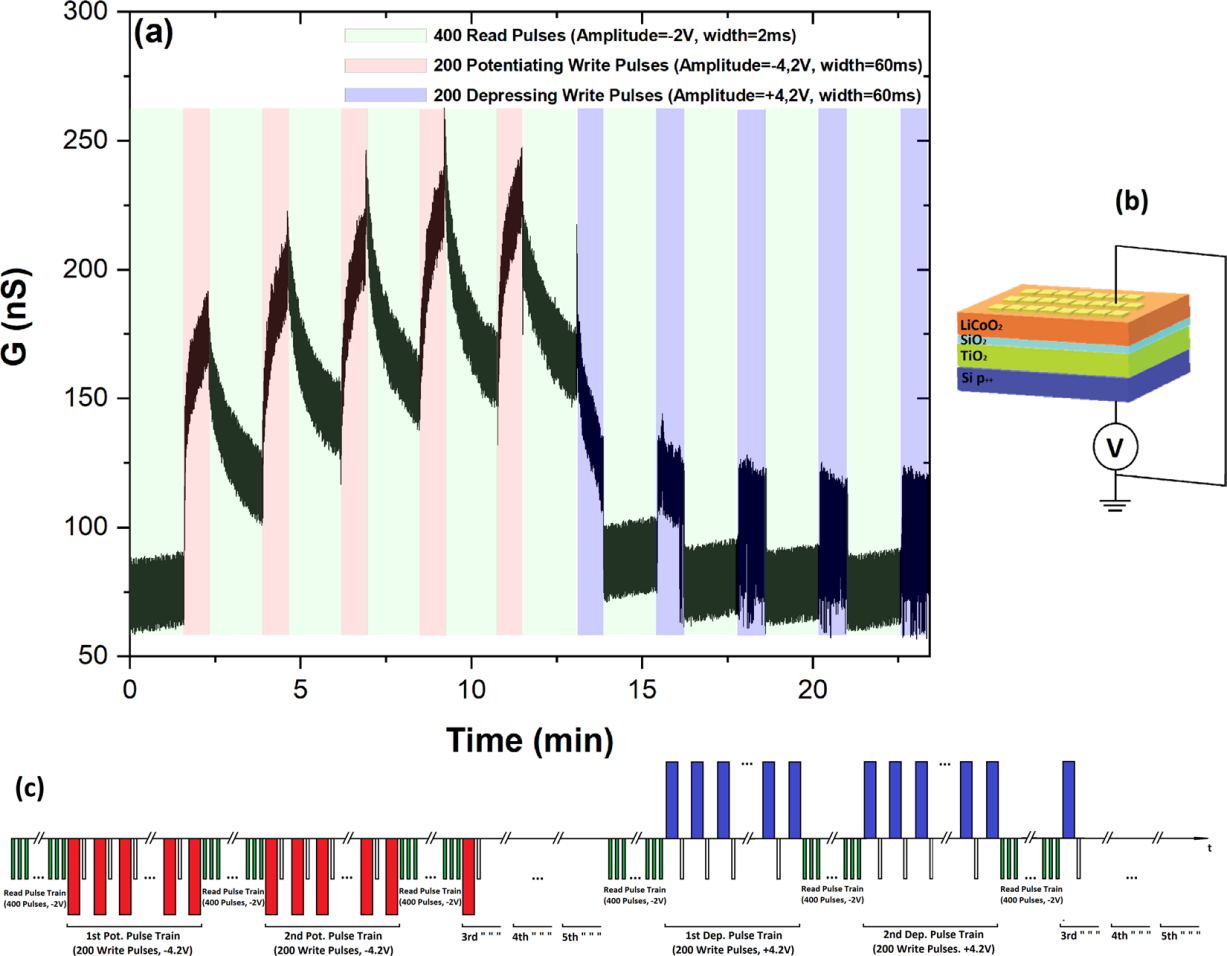
Conductance enhancement, relaxation and depression during voltage pulse stimulation. Pulsed stimulation, of the Au/Li_x_CoO_2_/SiO_x_/TiO_2_/p^++^-Si device with potentiating and depressing voltage pulse trains (write: -/+4.2 V, pulse width: 60 ms) while monitoring the conductance modulation (read: −2 V: pulse width: 2 ms), (top Au electrode: 300 × 300 μm^2^). Stimulated enhancement and subsequent conductance relaxation along with stimulated conductance depression can be observed (**a**). Schematics of the measurement setup (**b**) and the read/write pulse sequence applied on the device (**c**) are also illustrated.

Voltage pulse stimulation experiments have been carried out in order to explore the potential integration of the investigated devices in spiking neural networks (SNN). Applying voltage pulse trains (−/+4.2 V, with 60 ms width (w) and 120 ms spacing (s)) to the bottom p^++^-Si electrode enabled the potentiation/depression of the device’s conductance. The presence of an EMF between −1.2 V and 1 V after a potentiating cycle, along with the rectification effect observed at positive bias (Fig. 2, inset (b)), has led to the employment of read voltage pulses of −2 V to monitor the current modulation after each potentiating or depressing pulse (Fig. 4).

The pulse characterization begins with 400 read pulses (−2 V, 2 ms width, 180 ms spacing) that monitor the initial conductance of the device (green region in Fig. 4a). This is followed by a potentiating sequence of write/read pulse trains consisting of two parts: (i) 200 potentiating write pulses (−4.2 V, 60 ms width, 120 ms spacing - red regions in Fig. 4a), with each potentiating write pulse followed by a read pulse (−2 V, 2 ms width) and (ii) 400 read pulses (green regions in Fig. 4a). The potentiating sequence is repeated five times and then followed by a similar-in-scheme depressing sequence, where potentiating write pulses are replaced with depressing write pulses (+4.2 V - blue regions in Fig. 4a), which is also repeated five times.

As can be observed in Fig. 4, the device has an initial low average conductance, of ~80nS. The current drastically increases upon the application of the potentiation pulse trains, growing exponentially. Upon the removal of potentiating pulses, the device conductance relaxes, following an exponential decay. Exponential current-decay can be ascribed to the backpropagation of Li^+^ ions from the TiO_2_ anode and SiO_x_ electrolyte and their intercalation back to the Li_x_CoO_2_ cathode, thereby gradually reducing the device conductance.

Relaxation of the conductance in a predictable exponential decay, due to the internal Li^+^-diffusion dynamics of the device, render these devices an inherent timing mechanism. The device’s diffusive dynamics are described by the conductance relaxation time constant (τ), which lies in the range of several seconds (Supplementary Information, Fig. S3), providing a large enough window for calculations with millisecond pulses. It is noted that with proper material selection and engineering of the solid electrolyte properties, it is possible to drastically modify the relaxation dynamics^29^, thus enabling the optimization of the programming characteristics depending on the memory or synaptic behavior needed to be emulated.

Similar to the first potentiation train, application and removal of a second potentiation train stimulates the enhancement of the device conductance, resulting in an even higher maximum current. Moreover, every subsequent incoming potentiation pulse train, temporally placed in the relaxation tail of the previous potentiation, causes a cumulative enhancement on the device conductance, resulting in a saw-like conductance oscillation. Additionally, the application of the first depression pulse train, after the last potentiation train and spontaneous conductance relaxation, results in the stimulated relaxation of the device conductance. The read train following the first depression train reveals a new state of enhanced conductance (~90nS), compared to the initial state of the device. The subsequent depression trains further reduce the conductance in a descending stepladder manner, returning the device closer to its initial conductance state. This behavior indicates that the device can be potentiated/depressed at various different conductance states, through the manipulation of the Li^+^ ion concentration in the cathode and anode of the device. It is worth noting that these devices exhibited enhanced durability (up to 150,000 read/write operations without failure) compared to previous device configurations, where the Si substrate was used as the anode layer.

### Activity-dependent plasticity

Considering the top and bottom electrodes (Au and Si, respectively) as pre- and post-synaptic neurons and the actively switching volume between them as the synapse, the conductance modulation of the two-terminal device under investigation can be considered as a stimulated synaptic weight variation. In this respect, the relaxation current after each potentiation, observed only for post-synaptic potentials (negative bias applied on the bottom Si electrode), that exponentially decays with a time constant of several seconds and actively contributes to the cumulative enhancement of the device conductance, can be contemplated as an excitatory post-synaptic current (EPSC).

The diffusive dynamics observed in the device, and expressed as an EPSC, can be ascribed to the nanobattery effect and specifically to the diffusion of Li^+^ ions from the TiO_2_ anode and SiO_x_ electrolyte back to the parent Li_x_CoO_2_ cathode, due to the generation of an EMF. This effect is analogous to the spontaneous weight relaxation, caused by the gradually decaying Ca^2+^ residual concentration in the pre-synaptic region, of biological neuron cells. Summation of EPSCs, through the cumulative increase of the Li^+^ ion concentration in the post-synaptic region (further to the residual Li^+^ ions previously migrated), biorealistically mimics the origin of the paired pulse facilitation (PPF) mechanism observed in biological neurons. PPF of action potentials, because of the summation of EPSCs, is greatly involved in the activity-dependent plasticity of biological neurons^40,41^.

Cumulative increase of the measured current during consecutive potentiating pulse trains can be described by a PPF behavior, where a successive pulse stimulates the device to an even higher conductance value, through the summation of EPSCs. PPF in the investigated two-terminal devices has a temporal dependence and its effect can be enhanced or suppressed when the pulse width of the consecutive write pulses/trains is reduced or increased respectively (Supplementary Information, Fig. S2).

**Figure 5:**
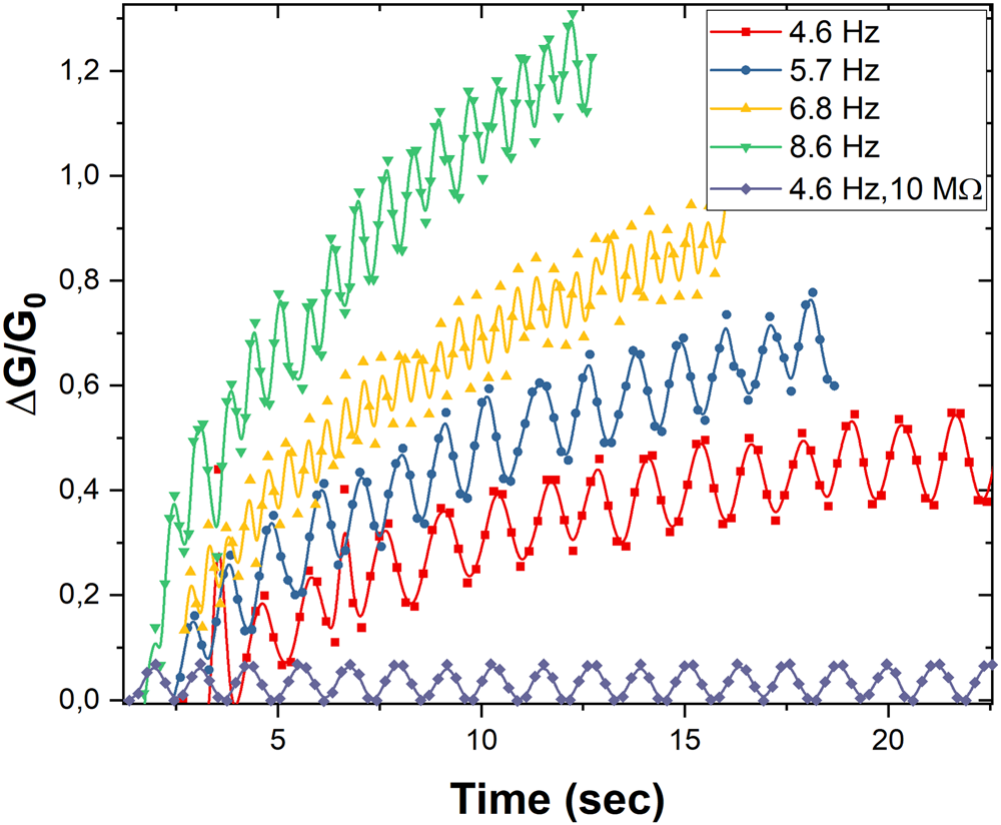
Frequency dependent plasticity effect. Dependence of the conductance variation over time on the stimulation frequency of the Au/Li_x_CoO_2_/SiO_x_/TiO_2_/p + +Si device (top Au electrode: 300 × 300 μm^2^). It is evident that the conductance variation increases with increasing stimulation frequency. Additionally, the conductance variation of a 10 MΩ resistor, stimulated with the same protocol, is depicted in order to elucidate that the observed conductance oscillations throughout the voltage pulse characterization are externally induced.

The combined effect of the PPF mechanism and the spontaneous EPSC decay, can be illustrated when the stimulation frequency is modulated. When 100 write (−4.2 V, 60 ms) pulses were applied on the device, the conductance variation (measured immediately after with read pulses of −2 V amplitude and 2 ms width) is enhanced with increasing stimulation frequency. The stimulation frequency was varied through the increase of spacing between write pulses (s = 20 ms, 50 ms, 80 ms, 120 ms). As shown in Fig. 5, increasing the stimulation frequency, results in a more pronounced impact on the device conductance. The stimulation frequencies appearing in the inset of Fig. 5 correspond to the real time rate of the applied write pulses.

The frequency dependence of the conductance enhancement is a manifestation of the equilibrium achieved between the Li^+^ ion accumulation in the TiO_2_ anode due to the potentiating pulses and the relaxation of the conductance due to the diffusion of Li^+^ ions from the TiO_2_ anode and SiO_x_ electrolyte to the Li_x_CoO_2_ cathode. Such an internal balancing between stimulated accumulation and spontaneous decay of Li^+^ ion concentration, provides a physical timing mechanism, rendering to these devices an inherent activity-dependent plasticity attribute.

Frequency-dependent synaptic plasticity, controlled by the Li^+^ ion concentration in the pre-synaptic region is analogous to the frequency-dependent plasticity of biological neurons, governed by the regulation of pre-synaptic Ca^2+^ concentration. Additionally, the ability of low frequency synaptic weight variation closely resembles the ability of chemical synapses to follow low frequency stimulations^42^.

In order to elucidate the origin of the conductance oscillation, evident in the pulse characterization measurements in this study, we applied the stimulation protocol described above on a resistor. A 10MΩ resisitor was selected because it roughly matches the series resisistance of the Au/Li_x_CoO_2_/SiO_x_/TiO_2_/p + +Si device. As can be seen in Fig. 5, application of the stimulation pulse train with alternating read (−2V) and write (−4V) pulses, results in a sinusoidal oscillation. As such, we can deduce that the oscillations of the measured current on the passive resistor element and the resistive switching devices during voltage pulse characterization are externally induced. The oscilations could possibly originate from the measurement configuration.

**Figure 6:**
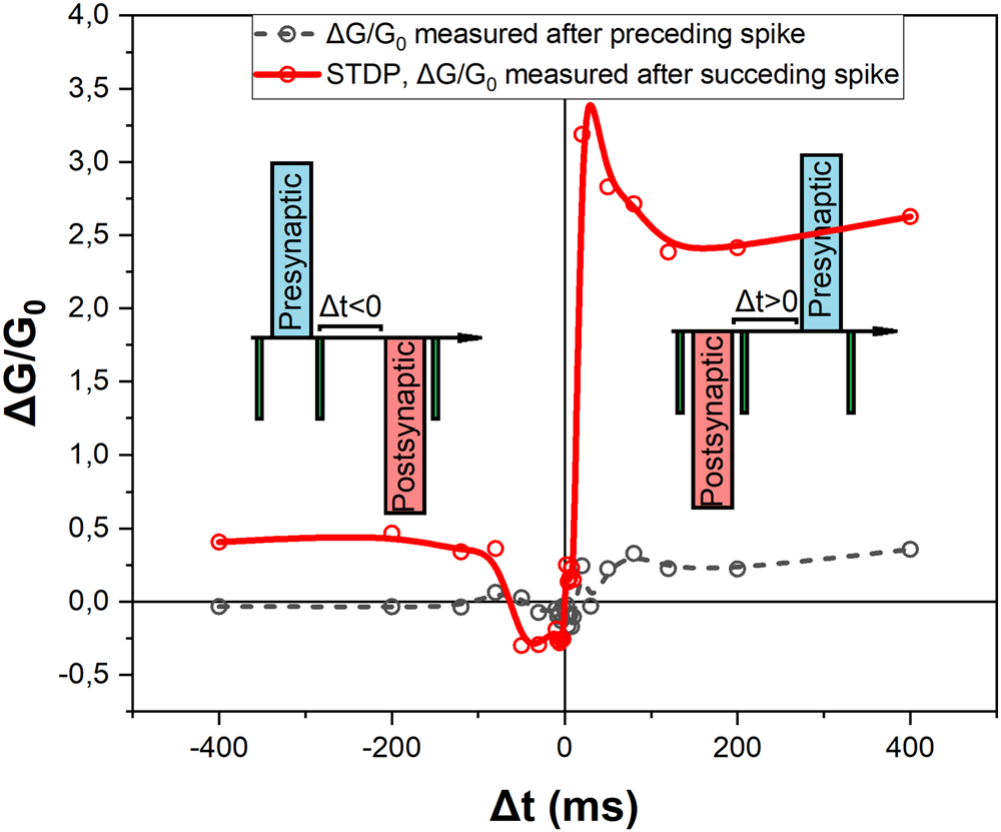
Spike-timing-dependent-plasticity (STDP) effect. When a pair of spikes (equal magnitude and opposite polarity, ±4.2 V, 300 ms) are sequentially applied on the two-terminal Au/Li_x_CoO_2_/SiO_x_/TiO_2_/p^++^-Si device (top Au electrode: 300 × 300 μm^2^) a STDP behavior is recorded (data corresponding to the red curve). Insets illustrate the presynaptic and postsynaptic spike sequence. Dependence of the conductance variation when a single postsynaptic spike (Δt > 0) or a single presynaptic spike (Δt < 0) is applied on the device is also depicted (data corresponding to the black curve).

Another manifestation of the impact of temporal association of pulses on the conductance modulation can be observed when a pair of potentiating and depressing pulses (spikes) are applied on the device (Fig. 6). Considering the investigated two-terminal device as a synapse, a pulse of −4.2 V applied to the bottom electrode was considered as a postsynaptic stimulation. On the other hand, a pulse of +4.2 V applied on the bottom electrode was considered as a presynaptic stimulation. While modifying the temporal spacing between postsynaptic and presynaptic spikes, the conductance response was monitored immediately after with read pulses (−2 V, 2 ms). A positive temporal difference (Δt > 0) is defined as the case where postsynaptic stimulation precedes presynaptic stimulation, while a negative temporal difference (Δt < 0) is defined as the reverse case (Fig. 6, insets).

By varying Δt, the spike-timing-dependent effect on the device conductance was monitored. A spike-timing-dependent-plasticity (STDP) behavior is observed between the device’s conductance variation (ΔG/G_0_, where ΔG = G_f_-G_0_) and the temporal association (Δt) between presynaptic and postsynaptic spikes (data corresponding to the red curve, Fig. 6). It is shown that with reducing Δt in either direction an emphasized effect on the potentiation or depression of the relative conductance results.

For negative temporal difference in the range of −60 ms to 0 ms, the cumulative effect of the pair of presynaptic/postsynaptic spikes lead to a conductance depression and the devices presented a negative conductance variation. While such a conductance reduction, below the stack’s initial conductance, would not be expected, it can possibly be explained by the Li^+^ ion loss effect discussed previously. As can be seen in Fig. 2, the first I-V cycles show a gradual decrease and an eventual stabilization of the maximum current, which was ascribed to Li^+^ ion accumulation inside the SiO_x_ electrolyte until a certain equilibrium concentration is reached. Therefore, the devices were stabilized prior to any other electrical characterization, using consecutive voltage sweeps. For temporal differences of −60 ms to 0 ms the leading positive voltage pulse possibly forces a fraction of the Li^+^ ions trapped inside the SiO_x_ electrolyte, to re-intercalate in the Li_x_CoO_2_ cathode reducing marginally the device’s conductance below its stabilized initial conductance.

In the range of −80 ms to −400 ms the device presented a positive conductance variation of roughly 0.5 (irrespective to the temporal difference of spikes), meaning that the synapse is relatively potentiated to G_f_ = 1.5G_0_, (Fig. 6, red curve). As can be seen, by the data corresponding to the black curve, for Δt > 0 the effect of a single postsynaptic spike on the conductance variation is very similar to that of a pair of presynaptic and postsynaptic spikes in the temporal range −80 ms < Δt < −400 ms. Thus, it is believed that for larger absolute temporal differences (for Δt < 0) the effect of the preceding presynaptic spike decays, and the device returns to its equilibrium state due to its internal diffusive dynamics, leaving only the effect of the succeeding postsynaptic spike to be reflected on the conductance variation.

Furthermore, for positive temporal differences the device presented a more pronounced enhancement of the conductance, compared to negative Δt, and this effect is enhanced with decreasing Δt, (Fig. 6, red curve Δt > 0). Increasing the temporal spacing between the preceding postsynaptic and succeeding presynaptic spikes, has a diminishing effect on the overall conductance enhancement due to the inherent diffusive dynamics involved in the relaxation of the conductance enhancement induced by the preceding postsynaptic spike.

More importantly, Fig. 6 indicates that, in addition to the dependence of the conductance state on Δt, there is a clear difference between applying a single spike and applying a pair of opposite spikes. While a single postsynaptic spike (black curve, for Δt > 0) slightly increases the device’s conductance, the introduction of a succeeding presynaptic spike leads to a more substantial enhancement through the cumulative effect of the spike pair similar to the synergistic effects of presynaptic and postsynaptic stimulations in biological synapses. Although the origin of this interesting synergistic effect is not clear at the moment, the mechanisms involved in the diffusive resistive switching Au/Li_x_CoO_2_/SiO_x_/TiO_2_/p^++^-Si two-terminal devices are actively investigated.

STDP behavior has been directly correlated to long-term potentiation of biological neurons^43,44^ and is actively involved in associative learning processes^45^. Forms of STDP learning algorithms are widely adopted in CMOS-based SNN computing paradigms, enabling the regulation of synaptic weights based on the temporal difference between pre-synaptic and post-synaptic spikes^46,47^, Moreover, STDP has been observed in several memristive systems with either conventional or diffusive behavior^48,49^. Implementation of diffusive-memristor-based STDP learning algorithms in SNN models has demonstrated an improved accuracy over conventional-memristor-based STDP. This is attributed to the ability of diffusive memristors to retain frequently transmitted information while forgetting less significant information, through their inherent weight decay dynamics^50^. Frequency-dependent plasticity and STDP response render the investigated two-terminal diffusive devices useful for direct emulation of activity-dependent bioinspired functionalities like adaptive synaptic plasticity, and the realization of unsupervised learning in SNNs.

## Conclusion

In an effort to reduce switching instabilities and limited durability observed in battery-like Au/LiCoO_2_/SiO_2_/p^++^-Si two-terminal devices, a thin TiO_2_ layer was introduced between the Si anode and the SiO_x_ electrolyte.

The Au/Li_x_CoO_2_/SiO_2_/TiO_2_/p^++^-Si two-terminal devices exhibited analog conductance modulation, based on the voltage-driven regulation of Li^+^ ion concentration in the cathode, reminiscent of the synaptic weight modulation according to the regulation of pre-synaptic Ca^2+^ concentration in biological neurons. Non-symmetric diode-like I-V characteristics observed in these devices allow the unidirectional signal propagation, similar to chemical or electrical (rectifying) biological synapses.

Furthermore, biorealistic synaptic functionalities, such as paired pulse facilitation – through the summation of excitatory post-synaptic currents – as well as frequency- and spike-timing-dependent plasticity, were facilitated by the inherent diffusive dynamics of the investigated two-terminal devices.

## Materials and Methods

Au/Li_x_CoO_2_/SiO_x_/TiO_2_/p^++^-Si devices were fabricated using a bottom-up approach, starting with the deposition of a Ti thin film on a p^++^-Si [(111), heavily doped: Boron, Resistivity: 0.001 − 0.003 ohm cm wafer] via DC sputtering. Subsequently, approximately 10 nm of SiO_x_ were deposited by reactive RF sputtering from a Si target. The SiO_x_/Ti/Si coated substrates were transferred to a pulsed laser deposition (PLD) chamber where the substrate temperature was raised to 600 °C and O_2_ background gas was backfilled. Ablation of a stoichiometric LiCoO_2_ target, using a pulsed UV Kr:F excimer laser (COMPexPro 201, *λ* = 248 nm and *τ* = 25 ns) operated at 1 Hz, with fluence of ~1.3 J/cm^2^ resulted in the deposition of approximately 45 nm of Li_x_CoO_2_ (x~0.99) (Li_x_CoO_2_) on the SiO_x_/Ti/Si substrates. During the deposition of Li_x_CoO_2_, the Ti metal thin film transformed to crystalline TiO_2_ (~30 nm), which served as the device’s new anode. The crystal structure and thickness of the individual films were investigated using Grazing-Incidence X-ray diffraction (GIXRD), X-ray reflectometry (XRR) (Rigaku SmartLab) and stylus profilometry (Bruker DektakXT), respectively.

The devices were completed with the deposition of Au electrodes (~100 nm thick) of various areas through a Ni shadow mask via DC sputtering, on top of the Li_x_CoO_2_/SiO_x_/TiO_2_/p^++^-Si stacks.

Electrical measurements were conducted at room temperature, in air, using a two-probe setup, sourcing voltage and measuring current. 20 μm-radius Be-Cu tips, controlled using micro-manipulators, were used to contact the top (Au) and bottom (Si) electrodes. A Keithley 6487 Picoammeter/Voltage Source, controlled by homemade LabVIEW routines (Virtual Instruments) via a GPIB bus, was employed to generate the excitation signals (voltage) and measure the device response (current). Current-voltage (I-V) characteristics of all cells were measured using voltage sweeps with maximum current compliance (25 mA) to enable observation of device behavior without artifacts. For pulse measurements, the programming signal (write pulses) consisted of customizable voltage spikes with respect to amplitude, length, and spacing. To avoid modification of the resistive state of the device, the cell response was measured using low-voltage spikes (read pulses) and simultaneous measurement of the current. Special attention was paid to the elimination of all delays in the sourcing and measurement of signals, other than those inherent in the software/hardware combination.

## Supplementary information

Supplementary Information.

## Data Availability

The datasets generated during and/or analysed during the current study are available in figshare repository, [http://doi.org/10.6084/m9.figshare.12251300].
