# Supplementary material for: Evidence of Biorealistic Synaptic Behavior in Diffusive Li-based Two-terminal Resistive Switching Devices
Source: Sci Rep. 2020 May 26;10:8711. doi: 10.1038/s41598-020-65237-0 (PMC7251090; doi:10.1038/s41598-020-65237-0)
Supplement: Supplementary file 1 — Supplementary Information. [file 41598_2020_65237_MOESM1_ESM.docx]

**Evidence of Biorealistic Synaptic Behavior in Diffusive Li-based Two-terminal Resistive Switching Devices**

**Panagiotis S. Ioannou^1^, Evripides Kyriakides^1^, Olivier Schneegans^2^, & *John Giapintzakis^1^**

^1^ Department of Mechanical and Manufacturing Engineering, University of Cyprus,

75 Kallipoleos Avenue, P.O. Box 20537, 1678 Nicosia, Cyprus

^2^ Laboratoire de Ge´nie E´lectrique et E´lectronique de Paris, CNRS, UPMC and Paris-Sud

Universities, Centralesup´elec, 11 rue Joliot-Curie, 91192 Gif-sur-Yvette, France

**Supplementary Information**

**Area dependence of the I-V characteristics.**

The two-terminal Au/Li_x_CoO_2_/SiO_x_/TiO_2_/p^++^-Si devices exhibited a dependence of the maximum current on the top Au electrode area that, in combination with the non-abrupt switching, indicate a homogeneous rather than a filamentary switching mechanism (Fig. S1).

**Figure S1.** I-V characteristics of the Au/Li_x_CoO_2_/SiO_x_/TiO_2_/p^++^-Si devices with different top Au electrode area (only the negative part of the sweeps are depicted). Inset shows the area dependence of the devices conductance measured at -5 V.

Applying voltage sweeps (-/+5 V at 0.1 V/s) on Au/Li_x_CoO_2_/SiO_x_/TiO_2_/p^++^-Si devices, with different top Au electrode area, resulted in qualitatively similar I-V characteristic curves, with maximum current and hysteresis area to be dependent on the top electrode area. Non-linear dependence of the maximum current on top electrode area (shown in the inset of Fig. S1) can be attributed to the device architecture adopted herein, which creates an asymmetry between the top and bottom electrode areas. Nevertheless, the abovementioned observations indicate a non-filamentary underlying mechanism, similar to that of Li^+^ ion batteries, based on the field-driven, homogeneous migration of Li^+^ ions from the Li_x_CoO_2_ cathode towards the TiO_2_ anode, in turn reducing the overall device resistance through the induced IMT in the Li_x_CoO_2_ cathode, due to the generated Li^+^ deficiency in Li_x_CoO_2_. While a non-filamentary resistive switching mechanism is possibly related to the increased stability of the Au/Li_x_CoO_2_/SiO_x_/TiO_2_/p^++^-Si devices compared to previous approaches (Au/Li_x_CoO_2_/SiO_x_/p^++^-Si), the reduction of maximum current with device miniaturization can potentially be promising for more power efficient switching with device scaling.

**Impact of the pulse width on the potentiation/depression.** In order to address the impact of the pulse width on the conductance modulation of the device, during pulsed potentiation/depression characterization, the width of the write pulses was varied (60, 80, 100, 120, 140, 160 ms), while the temporal spacing between successive write pulses was kept constant at 120 ms (Fig. S2).

**Figure S2.** Pulsed potentiation/depression characterization of the Au/Li_x_CoO_2_/SiO_x_/TiO_2_/p^++^-Si devices (Read: -2V, 2ms. Write: -/+4.2V, 60ms, 80ms, 100ms, 120ms, 140ms, 160ms. Spacing: 120ms), (Top electrode area: 300x300μm^2^).

As can be seen in Fig. S2, the write pulse width increase has a more pronounced impact on the potentiation of the device and at the same time enhances the separation between states, enabling a clearer observation of the depression of the device in “stepladder” manner during positive write pulse trains application.

In order to examine the relaxation dynamics of the devices for increasing write pulse width – after the first potentiation train is applied and during read-only operations – fitting was performed on the data. With a simple exponential decay function ($I={I_{o}+A}_{o}e^{-\frac{t}{\tau}}$), a relaxation time constant of ~30 s was extracted, irrespective of the write pulse width applied on the potentiation (Figs. S3 and S4). This could be indicative of the internal kinetics governing the Li^+^ ion diffusion due to the generated EMF, leading to the conductance relaxation of the device expressed as an EPSC. Additionally, as can be clearly observed in Fig. S4, upon increasing the write pulse width, the measured current during potentiation reaches a higher maximum value, as a result of higher Li^+^ ion accumulation achieved in the anode side. For write pulses wider than 120 ms, the current abruptly decays back to values observed for lower pulse widths. Specifically, while the maximum current value of the “relaxation tail” steadily increases for increasing write pulse width (60 ms, 80 ms and 100 ms), higher pulse width stimulation (120 ms, 140 ms and 160 ms) does not increase further this value. This can be indicative of the inability of the solid electrolyte used herein to withstand the increasing EMF for higher Li^+^ ion accumulation in the anode side, leading to the abrupt conductance relaxation of the device to a steady state limit. Occurrence of such a limit indicates that a maximum Li^+^ ion accumulation degree exists for the Au/Li_x_CoO_2_/SiO_x_/TiO_2_/p^++^-Si devices, which can be ascribed to the properties of the SiO_x_ solid electrolyte. Nevertheless, proper solid electrolyte engineering and material selection is expected to mitigate this limitation, consequently enabling the engineering of the relaxation characteristics of such two-terminal battery-like devices.


**Figure S3.** Exponential decay fitting of the current “relaxation tail” measured for stimulation of the Au/Li_x_CoO_2_/SiO_x_/TiO_2_/p^++^-Si device with 60 ms (-4.2 V) write pulses. A relaxation time constant ~30 s was extracted.

**Figure S4.** Device potentiation and subsequent exponential relaxation of the measured current, for increasing write pulse widths (Read: -2 V, 2 ms. Write: -4.2 V, 60 ms, 80 ms, 100 ms, 120 ms, 140 ms, 160 ms. Spacing: 120 ms). Although the measured current during potentiation reaches a higher maximum value for increasing pulse width stimulation, pulses wider than 120 ms have no further impact on the achieved steady state, due to limitation set by the SiO_x_ solid electrolyte.
